# Elevated LRRK2 autophosphorylation in brain-derived and peripheral exosomes in LRRK2 mutation carriers

**DOI:** 10.1186/s40478-017-0492-y

**Published:** 2017-11-22

**Authors:** Shijie Wang, Zhiyong Liu, Tao Ye, Omar S. Mabrouk, Tyler Maltbie, Jan Aasly, Andrew B. West

**Affiliations:** 1 Department of Neurology, Center for Neurodegeneration and Experimental Therapeutics, University of Alabama at Birmingham, Birmingham, AL 35233 USA; 2 Biogen, Discovery and Early Development Biomarkers, Cambridge, MA 02142 USA; 3 Dept of Neurology, Dept of Neuroscience, St. Olavs Hospital, Norwegian University of Science and Technology, 7030 Trondheim, Norway

**Keywords:** Park8, dardarin, Neurodegeneration, Movement Disorders, Biomarkers, Microvesicles

## Abstract

Missense mutations in the *leucine-rich repeat kinase 2* (*LRRK2*) gene can cause late-onset Parkinson disease (PD). *LRRK2* mutations increase LRRK2 kinase activities that may increase levels of LRRK2 autophosphorylation at serine 1292 (pS1292) and neurotoxicity in model systems. pS1292-LRRK2 protein can be packaged into exosomes and measured in biobanked urine. Herein we provide evidence that pS1292-LRRK2 protein is robustly expressed in cerebral spinal fluid (CSF) exosomes. In a novel cohort of Norwegian subjects with and without the G2019S-*LRRK2* mutation, with and without PD, we quantified levels of pS1292-LRRK2, total LRRK2, and other exosome proteins in urine from 132 subjects and in CSF from 82 subjects. CSF and urine were collected from the same morning clinic visit in 55 of the participants. We found that total LRRK2 protein concentration was similar in exosomes purified from either CSF or urine but the levels did not correlate. pS1292-LRRK2 levels were higher in urinary exosomes from male and female subjects with a *LRRK2* mutation. Male *LRRK2* mutation carriers without PD had intermediate pS1292-LRRK2 levels compared to male carriers with PD and controls. However, female *LRRK2* mutation carriers without PD had the same pS1292-LRRK2 levels compared to female carriers with PD. pS1292-LRRK2 levels in CSF exosomes were near saturated in most subjects, ten-fold higher on average than pS1292-LRRK2 levels in urinary exosomes, irrespective of *LRRK2* mutation status or PD diagnosis. These results provide insights into the effects of *LRRK2* mutations in both the periphery and brain in a well-characterized clinical population and show that LRRK2 protein in brain exosomes may be much more active than in the periphery in most subjects.

**Electronic supplementary material:**

The online version of this article (10.1186/s40478-017-0492-y) contains supplementary material, which is available to authorized users.

## Background

Genetic variation in the *leucine-rich repeat kinase 2* (*LRRK2*) gene associates with Parkinson disease (PD) susceptibility [21, 34]. Rare pathogenic missense mutations in exons encoding the GTPase domain (termed ROC) and linking COR (C-terminal of ROC) domains strongly predispose to late-onset PD [29]. The more common mutation in the kinase domain that may be present in 0.1% of Western populations, G2019S, has more variable and typically lower penetrance [32]. Lifetime risk for PD in G2019S carriers may be 80% in North African Berbers but as low as 20-30% in Ashkenazi Jews [17, 20, 29]. Studies analyzing Aβ, tau, and α-synuclein proteins in cerebral spinal fluid (CSF) from LRRK2 mutations carriers have identified nominal differences compared to idiopathic PD [1, 30]. Genome-wide association studies have identified common polymorphisms in *LRRK2* that associate with idiopathic PD, implicating LRRK2 function in susceptibility to late-onset PD in individuals without pathogenic mutations [19]. Few studies have yet biochemically analyzed LRRK2 protein in clinical samples from individuals with LRRK2 mutations or in the general idiopathic PD population.

In model systems, pathogenic *LRRK2* mutations increase the proportion of protein that is autophosphorylated [33], particularly at the serine 1292 residue [23]. We and others have demonstrated that LRRK2 kinase activity, and autophosphorylation at the 1292 residue, is required for LRRK2-linked neurotoxicity [11, 25, 26, 33]. The high frequency of *LRRK2* mutations in PD, particularly the G2019S mutation with incomplete penetrance, presents a unique opportunity to understand how surrogates of LRRK2 kinase activity like autophosphorylation may predict and drive PD progression [2, 13]. Previously, we demonstrated in urinary exosomes that the ratio of phosphorylated pS1292-LRRK2 to total LRRK2 is increased in male G2019S-*LRRK2* mutation carriers of Ashkenazi Jewish descent [6]. However, *in vitro* evidence suggests LRRK2 kinase activity stabilizes LRRK2 expression so that the ratio of pS1292-LRRK2 to total LRRK2 may not capture the overall increase in pS1292-LRRK2 protein [24]. Indeed, in a larger sample series of idiopathic PD from Birmingham, Alabama, we found that pS1292-LRRK2 levels are closely correlated with total LRRK2 levels when normalized to exosome housekeeping proteins [8]. Several studies have demonstrated increases in LRRK2 protein in frontal cortex post-mortem tissue from idiopathic PD and control [4, 5]. There are no previous studies evaluating pS1292-LRRK2 protein in the brain in clinical populations or in post-mortem studies. As LRRK2 kinase inhibitors move forward to the clinic, the characterization of kinase-activity surrogates like pS1292-LRRK2 in the brain may help identify subjects most likely to benefit from reductions of LRRK2 kinase activity [32].

Here, we quantify the absolute amount of pS1292-LRRK2 in CSF and urine from a novel cross-sectional cohort collected exclusively in Trondheim, Norway, focusing on G2019S-*LRRK2* mutation carriers. We measured pS1292-LRRK2, total LRRK2, and housekeeping exosomal proteins in both urine and CSF collected between 8 and 9:00 AM in (overnight) fasting Norwegians. Male and female subjects with or without PD, with or without the G2019S-*LRRK2* mutation, were evaluated. We found elevated pS1292-LRRK2 levels in urinary exosomes from mutation carriers versus controls. Strikingly, pS1292-LRRK2 levels were near saturated in most *LRRK2* mutation carriers in exosomes isolated from the CSF. These results provide the first measurements of brain-derived LRRK2 protein in clinical samples. While we could not distinguish *LRRK2* mutation carriers from controls in CSF due to saturation of the pS1292-LRRK2 phosphorylation site in many of the subjects, our results further link the G2019S-*LRRK2* mutation with upregulated kinase activity.

## Methods

### Clinical cohort and biospecimens

All protocols were approved by the local Institutional Review Boards. Participants were recruited in the outpatient clinic of the Department of Neurology, St. Olavs hospital, Trondheim, Norway. Urine specimens were collected for this study from 132 participants that included healthy controls (LRRK2- PD-), age-matched G2019S-*LRRK2* mutation carriers without manifest PD (LRRK2+ PD-), G2019S-*LRRK2* mutation carriers with PD (LRRK2 + PD+), and non-*LRRK2* mutation carriers with PD (i.e., idiopathic PD, LRRK2- PD+, see Table 1). CSF specimens were collected from 82 participants (Table 2). Of these subjects, 55 donated both urine and CSF in the same clinic visit and were analyzed here (Table 3). All lumbar punctures were performed between 8 and 9 AM on overnight fasting subjects, and urine samples were collected thereafter to around 10 AM. A small amount of CSF was immediately sent for cell counts and tests for glucose and proteins. Urine and CSF was centrifuged within 15 minutes after the end of collection, aliquoted, and biobanked.

**Table 1:** Clinical data and demographics corresponding to urine samples from subjects with or without G2019S-*LRRK2* mutations, with or without PD

	LRRK2+/PD-	LRRK2+/PD+	LRRK2-/PD+	LRRK2-/PD-	*p* value^a^
Gender M(F)	29(30)	8(18)	23(16)	2(6)	ns
Age	60 (52-66)	59 (53.75-70.25)	59 (53-63)	60.5 (56-75)	ns
Age at onset	NA	55 (47-60.25)	55 (50-60)	NA	ns
LEDD	NA	500 (400-650)	300 (0-600)	NA	ns
UPDRS_III	4 (0-6)	19.5 (15.75-22.25)	19 (16.5-24)	0 (0-0)	<0.0001
MoCA	26 (26-29)	26 (25-27)	28 (26-28)	27 (<26-30)	ns
H&Y	0 (0-0)	2 (2-2.5)	2 (2-2)	0 (0-0)	<0.0001

*LEDD* is L-dopa equivalent daily dosage, *UPDRS* is Unified Parkinson’s Disease Rating Scale, *MoCA* is Montreal Cognitive Assessment, *H&Y* is Hoehn & Yahr scale, *NA* is not applicable; Values reported are group median (interquartile range). ^a^Determined by comparing LRRK2+/PD- to LRRK2+/PD+ using the Dunn post hoc test following Kruskal-Wallis test of all groups. *ns* is not significant as determined by Pearson chi square (gender and MoCA), ANOVA (age at test), t test (age at onset), Mann-Whitney test (LEDD) and Kruskal-Wallis test (H&Y, UPDRS III)

**Table 2:** Clinical data and demographics corresponding to CSF samples from subjects with or without G2019S-*LRRK2* mutations, with or without PD

	LRRK2+/PD-	LRRK2+/PD+	LRRK2-/PD+	LRRK2-/PD-	*p* value^a^
Gender M(F)	13(26)	3(16)	11(8)	0(5)	0.008
Age	63 (56-69)	57 (53-69)	60 (50-67)	60 (44.5-74)	ns
Age at onset	NA	55 (47-65)	57 (50-60)	NA	ns
LEDD	NA	600 (250-675)	300 (0-550)	NA	ns
UPDRS_III	5 (0-6)	18 (15-22)	20 (16.25-22)	0 (0-0)	<0.0001
MoCA	26 (26-28)	26 (25-27)	27 (26-28)	27 (<26-30)	ns
H&Y	0 (0-0)	2 (2-2.5)	2 (2-2)	0 (0-0)	<0.0001

*LEDD* is L-dopa equivalent daily dosage, *UPDRS* is Unified Parkinson’s Disease Rating Scale, *MoCA* is Montreal Cognitive Assessment, *H&Y* is Hoehn & Yahr scale, *NA* is not applicable; Values reported are group median (interquartile range). ^a^Determined by comparing LRRK2+/PD- to LRRK2+/PD+ using the Dunn post hoc test following Kruskal-Wallis test of all groups. *ns* is not significant as determined by Pearson chi square (MoCA), ANOVA (age at test), t test (age at onset), Mann-Whitney test (LEDD) and Kruskal-Wallis test (H&Y, UPDRS III)

**Table 3:** Clinical data and demographics corresponding to subjects who donated both CSF and urine in the same visit

	LRRK2+/PD-	LRRK2+/PD+	LRRK2-/PD+	LRRK2-/PD-	*p* value^a^
Gender M(F)	11(15)	3(12)	5(6)	0(3)	ns
Age	62.5 (56-67.25)	58 (53-69)	57 (50-64)	73 (60-75)	ns
Age at onset	NA	55 (47-65)	57 (50-60)	NA	ns
LEDD	NA	600 (375-662.5)	400 (300-600)	NA	ns
UPDRS_III	4.5 (0-6)	18 (15-20)	18 (16-22.5)	0 (0-0)	<0.0001
MoCA	26 (26-28)	26 (25-27)	27 (26-28)	26 (<26-30)	0.01
H&Y	0 (0-0)	2 (2-2.5)	2 (2-2)	0 (0-0)	<0.0001

*LEDD* is L-dopa equivalent daily dosage, *UPDRS* is Unified Parkinson’s Disease Rating Scale, *MoCA* is Montreal Cognitive Assessment, *H&Y* is Hoehn & Yahr scale, *NA* is not applicable; Values reported are group median (interquartile range). ^a^Determined by comparing LRRK2+/PD- to LRRK2+/PD+ using the Dunn post hoc test following Kruskal-Wallis test of all groups. *ns* is not significant as determined by Pearson chi square (gender), ANOVA (age at test), t test (age at onset), Mann-Whitney test (LEDD) and Kruskal-Wallis test (H&Y, UPDRS III)

Urine and CSF samples were processed and analyzed by an investigator blinded to their identify. CSF hemoglobin level was determined using ELISA method (Bethyl Labs, #E88-134). Urine was assessed for leukocyte count, pH, glucose, total protein, red-blood cells, and specific gravity via test strips (Fisher). All participants were clinically evaluated for PD severity using the Unified Parkinson’s Disease Rating Scale (UPDRS) part III, Hoehn & Yahr scale, modified Schwab & England scale, Montreal Cognitive Assessment (MoCA), and L-dopa equivalent daily dose (LEDD) calculated as described [8].

### Exosome isolation and characterization

Supernatants from cell cultures were centrifuged 10,000 x g at 4 °C for 30 min, supernatant and centrifuged at 100,000 x g for 1 hour at 4°C, and resultant pellets lysed in SDS buffer (2% SDS, 10% glycerol, 60 mM Tris-CL, pH 6.8, and 5% dithiothreitol). HEK293 cells and primary macrophages were cultured and transfected with LRRK2 plasmids as previously described [7, 18]. Urine, CSF, or serum (Supplemental) samples were quick-thawed in a shaking 42 °C water bath and placed on ice after thawing. Samples were centrifuged at 10,000 x g at 4 °C for 30 min, supernatant centrifuged at 100,000 x g for 1 hour at 4 °C, and the resultant exosome-enriched pellet was washed in 1 mL saline and centrifuged at 100,000 x g for one hour at 4 °C. Randomly selected exosome-enriched pellets were analyzed via single-particle tracking Nanosight analysis. Representative vesicle size and concentration traces were recorded over five runs (60 sec per run) each and analyzed with NTA software (Nanosight).

### Quantification of pS1292-LRRK2 in recombinant protein standards by LC-MS

Full-length recombinant Flag-G2019S-LRRK2 protein was purchased from Invitrogen and purity was assessed by Coomassie-stain SDS-PAGE. Concentrations of LRRK2 protein were assessed by BCA assay (Pierce). LRRK2 protein (100 nM) was incubated with 200 μM ATP in buffer containing 150 mM NaCl, 50 mM Tris-HCl, and 10 mM MgCl_2_ at 37 °C for up to 60 min. LRRK2 protein standards were digested with endoproteinase Glu-C (Roche) in 50 mM Tris-HCl (pH7.0) at 37 ^0^C overnight, and heavy isotope labeled peptides MGK^LSKIWDLPLDE and MGK^L(pS)KIWDLPLDE (New England Peptide, K^: ^13^C_6_^15^N_2_-lysine) were added to samples that were injected into a 300 μm x 5 mm Thermo μ-Precolumn (C18 Pepmap 100, 5 μm, 100 Å pore). After washing with 0.1% formic acid and 2% acetonitrile in water at 20 μL per min, 35 ^0^C, for 4.5 min, samples were separated on a 75 μm x 150 mm Thermo EASY-Spray column (C18 Pepmap, 3 μm particle, 100 Å pore) using a Thermo Ultimate 3000 RSLCnano LC system. Mobile phase A consisted of 0.1% formic acid in water and mobile phase B consisted of 0.1% formic acid in acetonitrile. The peptides were eluted using a linear gradient from 2% to 90% mobile phase B at 400 nL per min, 40 ^0^C, over 15.5 min. The phospho and non-phospho S1292-containing peptides and their internal standards were detected with a Thermo Q Exactive plus Orbitrap mass spectrometer running PRM (parallel reaction monitoring). The electrospray voltage, capillary temperature, and S-lens RF were 2.0 kV, 300 ^0^C, and 50 V, respectively. The b12 and b13 fragments (m/z values as indicated below) of the peptides were summed to generate ion chromatograms, and ratios between the peak areas of the light and heavy peptide were used for quantifying the phospho and non-phospho S1292 peptides from recombinant LRRK2 protein to calculate the degrees of phosphorylation. Detection limit for the unphosphorylated peptide (MGKLSKIWDLPLDE) was estimated to be approximately 100 pg mL^-1^ while it was approximately 300 pg mL^-1^ for the phosphorylated peptide (MGKLpSKIWDLPLDE).

**Table:** 

Detection peptide	type	Precursor ion (*m/z)*	Product ion (*m/z)*	NCE (V)
MGKLSKIWDLPLDE	Surrogate peptide	548.96	691.8969 (b12) 749.4103 (b13)	15
MGKLSKIWDLPLDE	Internal standard	551.63	695.9040 (b12) 753.4174 (b13)	15
MGKL(pS)KIWDLPLDE	Surrogate peptide	575.62	731.8800 (b12) 789.3935 (b13)	14
MGKL(pS)KIWDLPLDE	Internal standard	578.29	735.8871 (b12) 793.4006 (b13)	14

### Quantification of proteins by immunoblot

To measure the amount of total LRRK2 and pS1292-LRRK2 in clinical samples, a pool was first created by combining 1/10 (w/v) of all samples from the experiment used in each analytical run. The amount of pS1292-LRRK2 and total LRRK2 protein in the pool sample was determined by comparing the pool together with recombinant protein analyzed by mass spectrometry to determine absolute values of total LRRK2 and pS1292-LRRK2 via immunoblot method (see Additional file 1: Figure S1 and S2). The amount of pS1292-LRRK2 and total LRRK2 protein in the HEK293 cell and exosome samples was determined similarly, with WT-LRRK2, pathogenic mutations G2019S, R1441G and double LRRK2 mutation R1441C/G2019S samples that covers the entire signal range of LRRK2 and comparing with recombinant protein previously analyzed by mass spectrometry. Samples were electrophoresed on 4–20% TGX gradient gels (BioRad) and transferred onto PVDF membranes (Immobilon-FL, Millipore). Membranes were blocked using 5% non-fat milk in TBST and cut in half for analysis of LRRK2 in the top half and exosomal housekeeper proteins Tsg101 or flotillin-1 in the bottom half. Digital signal intensities were recorded with the ChemiDoc Touch platform and ImageLab software (BioRad). Linearity of digital signals were confirmed across the possible dynamic range (10-600pg depending on antibody conditions, see Additional file 1: Figure S1 and S4).

The following antibodies were used: N241A/34 anti-LRRK2 (Antibodies Inc), MJFF2 c41-2 anti-LRRK2 (Abcam), UDD3 anti-LRRK2 (Abcam), UDD2-10 anti-pS935 LRRK2 (Abcam), MJFR-19-7-8 anti-pS1292-LRRK2, UDD3 anti-LRRK2 (Abcam), D2V7J anti-flotillin-1 (Cell Signaling), and polyclonal antibodies to phospho-threonine (P-Thr-Poly 9381, Cell Signaling) and Tsg101 (ab30871, Abcam).

### Phosphatase treatment of membranes

For some immunoblots, after transfer of protein was completed, membranes were blocked with 3% BSA (Jackson ImmunoResearch) in TBS-T and treated with or without 250 units mL^-1^ calf intestinal alkaline phosphatase (CIP, New England BioLabs) in CutSmart buffer (New England BioLabs) for 2 hours at 37 ^0^C. Quantification of proteins on the membranes then proceeded as described above.

### Statistics and figures

All samples were assayed and quantified in randomized order and group assignments were made after final data curation. One-way ANOVAs with Tukey post hoc test, or unpaired t-test, was used for parametric continuous variables to compare group means. Kruskal-Wallis one-way analysis with Dunn test for post hoc comparison, or Mann-Whitney U test, were used to compare median values of categorical and nonparametric variables. Correlation analyses were performed using Spearman Rank-Order tests to accommodate nonparametric variables. All significance levels are reported as two-tails, corrected for multiple testing, with *p* <0.05 interpreted as significant. Statistical analysis was performed using JMP Pro 10 (SAS Institute Inc), graphs built with GraphPad Prism 5.0 and arranged in Adobe Illustrator 10.

## Results

### Exosome pS1292-LRRK2 levels correlate with cellular LRRK2 kinase activity

**Fig. 1:**
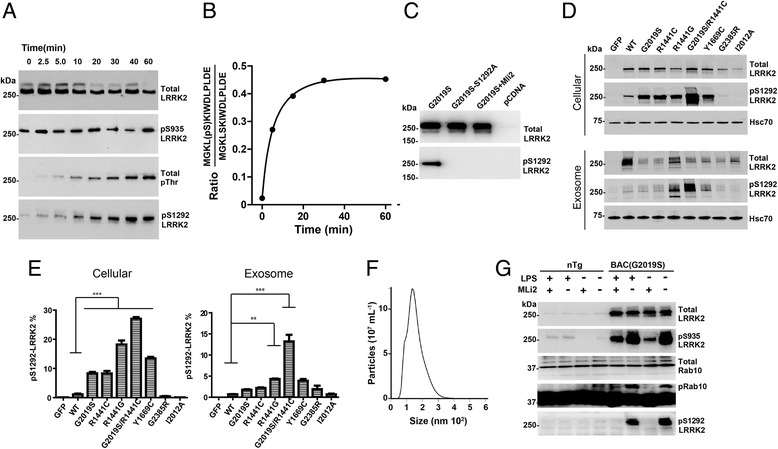
Generation of recombinant pS1292-LRRK2 standards and correlation of exosome and cellular pS1292-LRRK2 levels. **a** Recombinant G2019S-LRRK2 protein was incubated in kinase reaction buffer for the indicated time. Representative immunoblots are shown. **b** LC-MS analysis of the pS1292 peptide isolated from recombinant protein from panel A. **c** Representative immunoblots of S1292A-LRRK2 protein, expressed in HEK293 cells, to validate the authenticity of pS1292-LRRK2 signal. **d** Representative immunoblots and quantification of HEK293 cell and exosomal lysates, 48-hours after transfection with the indicated LRRK2-encoding plasmids. eGFP-only controls, WT-LRRK2, pathogenic mutations G2019S, R1441C/G, Y1699C, and the double-LRRK2 mutation G2019S/1441C are indicated. Exosome-depleted cell media were incubated with the transfected cells for 24-hours prior to isolation. **e** Calculated pS1292-LRRK2 levels for cellular and exosome fractions of total LRRK2 protein. Column graphs show mean values with S.E.M. as error bars representing three independent experiments. ***p-value<0.001, **p-value<0.01, *p-value<0.05, ns: *p*-value>0.05. *p* value between groups were calculated using Tukey’s multiple comparison test. **f** Representative nanoparticle tracking analysis of the exosomes isolated from the HEK293 cell cultures in panel C. **g** Bone marrow derived macrophages from either non-transgenic (Ntg) or G2019S-*LRRK2* transgenic mice were treated with LPS to increase pS935-LRRK2 levels, or the LRRK2 kinase inhibitor MLi2 (100 nM) to decrease phospho-LRRK2 levels, as indicated. Rab10 phosphorylation was assessed via phos-tag analysis as described [26]

Previously, we demonstrated that heterozygous G2019S-*LRRK2* mutations increased the ratio of pS1292-LRRK2 to total LRRK2 protein ~4-fold in urinary exosomes of G2019S carriers as compared to non-carriers [6]. These and other measurements from model systems have not revealed the proportion of LRRK2 protein phosphorylated at pS1292, as only relative ratios of signal have been presented in past studies [6, 8, 14, 23]. Further, it is not known how different *LRRK2* mutations and different cellular and exosome sources may differ in the amount of pS1292-LRRK2 with respect to other LRRK2 kinase substrates like Rab10 [26]. In measuring pS1292-LRRK2 from *in vitro* LRRK2 kinase reactions, we observed a low-starting level of pS1292-LRRK2 in recombinant G2019S-LRRK2 protein that increased over time, as expected (Fig. 1a). In contrast, the constitutive phosphorylation site pS935 that is not a LRRK2 autophosphorylation site did not change over time. Other threonine LRRK2 autophosphorylation sites have been described in the LRRK2 ROC GTPase domain [10, 12, 31]. Cumulatively, these increased in abundance over time like pS1292 as revealed by pan-phospho-threonine detection (Fig. 1a). Thus, *in vitro*, pS1292-LRRK2 levels correlate closely with overall autophosphorylation. Using a novel mass spectrometry approach to quantify pS1292-LRRK2 over time in recombinant protein, we could determine that 45% of the LRRK2 protein was pS1292-LRRK2 after one-hour (Fig. 1b). Cellular levels of immunoprecipitated pS1292-LRRK2 (before kinase assays) demonstrated only ~2% of total LRRK2 is pS1292-LRRK2 (Fig. 1a, b). To verify the specificity of the pS1292-LRRK2 antibody for a phosphorylated peptide, we introduced a Ser1292Ala mutation into the G2019S-LRRK2 backbone and found that the Ser1292Ala mutation blocks pS1292-LRRK2 antibody binding (Fig. 1c).

We next compared the effect of pathogenic LRRK2 mutations on pS1292-LRRK2 levels in both cell extracts from transfected HEK293 cells as well as extracellular exosomes purified from their cell culture media. As expected, all the pathogenic LRRK2 mutations increased cellular levels of pS1292-LRRK2 (Fig. 1d), with the highest levels induced by a double-mutation R1441C / G2019S, also known to enhance neurotoxicity in primary neurons [23]. pS1292-LRRK2 levels were similar between WT-LRRK2, the prevalent Far-East Asian G2385R-LRRK2 risk variant, and the non-pathogenic I2012A-LRRK2 variant. Through comparison to recombinant pS1292-LRRK2 standards defined by mass spectrometry (and in linear range, see Additional file 1: Figure S2), we deduced the percent of pS1292-LRRK2 in cells in the double-LRRK2 mutant as ~27% of available LRRK2 protein (Fig. 1d,e).

Exosome protein levels do not usually reflect the levels of proteins and their post-translational modifications found in cell cytosols due to the mechanisms that select for exosome cargo and transport in the endolysosomal system [28]. However, exosomes from the transfected HEK293-derived exosomes revealed similar pS1292-LRRK2 levels as compared to the parental cellular lysates (Fig. 1d, e). The effects of the double-mutation G2019S/R1441C appeared slightly exaggerated compared to other mutations, and in general, pS1292-LRRK2 levels were slightly lower on average (Fig. 1e). Analysis of the exosome pellets from these cells via single-particle nanotracking revealed a typical microvesicle population that had an average size of 134 nm (Fig. 1f). Thus, in these cells, exosomes reported pS1292-LRRK2 levels that were consistent with cellular levels that were increased by pathogenic *LRRK2* mutations.

In humans, *LRRK2* expression is highest in circulating leukocytes in the blood according to RNAseq tissue expression profiles [32]. Leukocytes like macrophages and monocytes may secrete exosomes to control some aspects of immune reactions [15]. To measure endogenous LRRK2 protein in macrophages in culture, we isolated exosomes and cellular lysates from wild-type or G2019S-LRRK2 expressing macrophages. In cellular lysates, we could not reliably detect any pS1292-LRRK2 in wild-type macrophages, whereas pS1292-LRRK2 levels were robust with G2019S-LRRK2 expression (Fig. 1g). pS1292-LRRK2 signal, as well as pRab10 signal (a *trans* LRRK2 kinase substrate), was ablated with one-hour treatment of a LRRK2 kinase inhibitor. However, we were unable to detect pS1292-LRRK2 or total LRRK2 protein in exosomes isolated from primary macrophages, irrespective of LPS stimulation. These results suggest that leukocytes like macrophages that express high LRRK2 levels may not be an important source for LRRK2 protein in exosomes.

### Brain-derived exosomes have elevated pS1292-LRRK2 levels

**Fig. 2:**
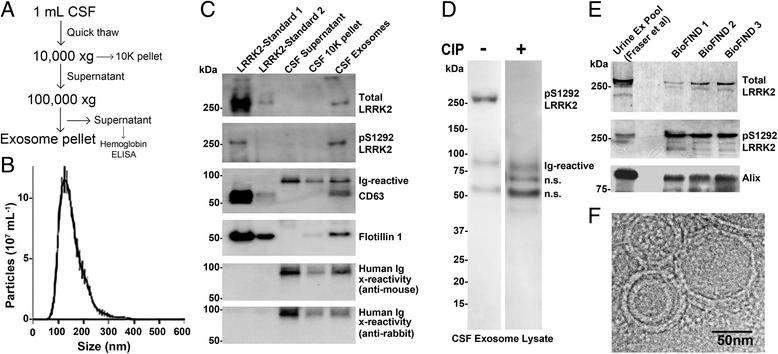
Isolation and characterization of brain-derived exosomes from CSF. **a** Exosomes were isolated with a two-step centrifugation. **b** Representative nanoparticle tracking analysis of the exosome-enriched fraction. Vesicle size and concentration traces were recorded and analyzed over five runs (60s per run) each. **c** Immunoblots of CSF exosome pellet lysates together with recombinant LRRK2 protein control spiked into HEK293 lysates. Recombinant LRRK2 protein was included at a 1:100,000 (w/v) ratio with HEK293 lysate in LRRK2-Standard 1, and 1:1,000,000 (w/v) ratio in LRRK2-Standard 2. Lysates were also probed with mouse and rabbit secondary antibody alone to confirm the identity of the bands that are due to cross reactive immunoglobulin that co-precipitated with the exosomes (Ig cross-reactive). **d** Phosphatase treatment of membranes prior to pS1292-LRRK2 detection in CSF exosome lysates. n.s. is not-specific, band of unknown identity. **e** Comparison of relative LRRK2 expression and pS1292-LRRK2 in purified urinary exosomes, and three representative CSF exosome lysates from the BioFIND cohort. **f** Representative electron microscopy image of the CSF exosome pellet, scale bar is 50 nm

Previously we detected total LRRK2 protein in exosome-enriched fractions purified from post-mortem remnant CSF [7]. To determine whether we can detect pS1292-LRRK2 in biobanked clinical CSF samples verified to have low or no detectable hemoglobin, we analyzed three samples from neurologically normal controls provided by the BioFIND repository. Guided by Nanosight analysis of microvesicle fractions, we applied a modified differential ultracentrifugation strategy to isolate an exosome fraction that harbors vesicles with an average size of ~125 nm (Fig. 2a, b). These exosomes were similar in characteristics to those isolated from HEK293 cells. Using a reference pS1292-LRRK2 protein, we detected appreciable signal for total LRRK2 and pS1292-LRRK2, but only in the exosome-enriched fraction and not in supernatants or low speed (10,000 x *g*) pellets (Fig. 2c). The signal elicited by the pS1292-LRRK2 antibody in the human CSF exosome pellet was destroyed by brief treatments of the membranes with calf intestinal alkaline phosphatase (CIP), indicating that the cross-reactive band is a phospho-protein and of the exact molecular weight as our full-length recombinant protein standard (~280 kDa, Fig. 2d). As opposed to HEK293, macrophage, and urinary exosome lysates, a significant immunoglobulin signal due to the species secondary antibody is apparent at ~90 kDa, as well as a low-intensity band (compared to pS1292-LRRK2) at ~70 kDa. In these CSF specimens, hemoglobin levels were below our ELISA detection limit (<2 pg mL^-1^), suggesting LRRK2 protein in the CSF is probably not from blood product contamination as our lower detection limit for pS1292-LRRK2 is higher than 2 pg mL^-1^.

In a recent past study, a ‘pooled’ exosome fraction was created that is composed of urinary exosome lysates from ~160 subjects with and without PD [8]. Using our analytical process described here, we can estimate ~5% of total LRRK2 in this pools sample is pS1292-LRRK2 (Fig. 2e). In comparing the BioFIND CSF exosome lysates, total LRRK2 protein was lower but pS1292-LRRK2 was much higher and calculated at ~80% on average (Fig. 2e). This level of pS1292-LRRK2 was higher than that we recorded from urinary exosomes from LRRK2 mutation carriers, higher than that we could achieve by *in vitro* kinase assays with recombinant protein, and higher than from HEK293 exosomes transfected with our double-LRRK2 mutation construct. Cryo-electron microscopy analysis of the CSF exosome pellets revealed a similar microvesicle constituency as our past observations with urinary exosomes [7] and single-particle tracking measurements here (Fig. 2f).

### Elevated pS1292-LRRK2 levels in Norwegian LRRK2 mutation carriers

Previously, we found an elevated ratio of pS1292-LRRK2 normalized to total LRRK2 protein and the control exosome protein Tsg101 in urinary exosomes isolated from male G2019S-*LRRK2* mutation carriers from the MJFF LRRK2 Cohort [6]. Here, we sought to determine whether a similar relationship exists in a Norwegian cohort of LRRK2 mutation carriers, with and without PD, and extended the analysis to include females as well as an absolute quantification of pS1292-LRRK2. The cohort we analyzed consisted of 132 subjects that donated urine (Table 1), 82 subjects that agreed to donate CSF (Table 2), and 55 subjects that donated both CSF and urine in the same clinic visit (Table 3).

**Fig. 3:**
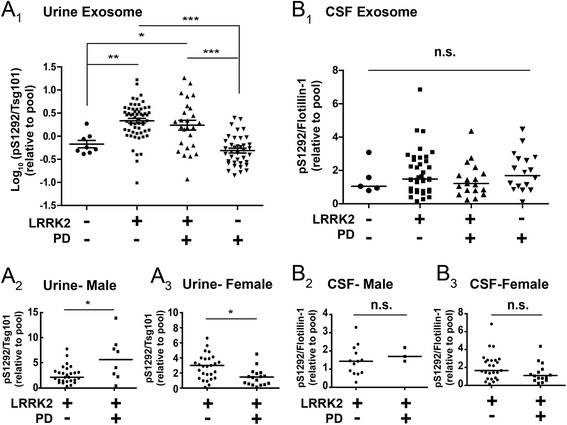
Quantification of exosomal pS1292-LRRK2 in urine and CSF (**a**) Scatter plots showing pS1292-LRRK2 expression levels normalized to TSG101 expression, relative to the pool (all samples, N=132). Bars depict mean values with error bars showing S.E.M. Quantifications were based on the average value of three independent immunoblot runs. **A**_**2,3**_ The LRRK2 mutation carrier groups (PD+/-) are broken further according to sex as indicated. Bars show median values. **b** Scatter plots showing relative pS1292-LRRK2 expression level normalized to flotillin-1 expression, relative to the pool (all samples, N=81). The LRRK2 mutation carrier groups (PD+/-) are broken further according to sex as indicated (**B**_**2,3**_). Bars show median values. ***p-value<0.001, **p-value<0.01, *p-value<0.05, ns: *p*-value>0.05. *p* value between groups were calculated using Tukey’s multiple comparison test (figure **a**_1_ and **b**_1_) and Mann-Whitney test (figure **a**_2-3_ and **b**_2-3_)

Measurements of pS1292-LRRK2 levels in urinary exosomes, normalized to the abundance of the control exosome protein Tsg101, revealed elevated pS1292-LRRK2 in G2019S-*LRRK2* mutation carriers compared to non-carriers (~4.8 fold on average, p<0.0001, Fig. 3a1). In breaking groups according to sex, male G2019S-*LRRK2* mutation carriers with PD had higher pS1292-LRRK2/Tsg101 levels than in carriers without PD (~8.9 fold versus ~3.8 fold, p=0.04). However, in the female group, this trend was reversed (~3.6 fold versus ~5.4 fold, p=0.012, Fig. 3a3). Thus, the female mutation carriers with PD had similar levels of pS1292-LRRK2 as the male carriers, but the age-matched female LRRK2 mutation carriers without PD also had high levels. We did not detect significant correlations between pS1292-LRRK2/Tsg101 levels and any urine characteristic we measured in the samples (leukocyte count, pH, glucose, total protein, red-blood cells, and specific gravity), or any demographic or clinical data (Spearman R values, all *p* values >0.1, Table 1).

In CSF exosome isolations from the cohort, we could not reliably detect the exosome protein Tsg101 whereas we could reliably measure the exosomal housekeeping protein flotillin-1 in the samples. Analysis of CSF for pS1292-LRRK2 levels, as normalized to the abundance of flotillin-1, revealed similar amounts in the groups irrespective of PD diagnosis, *LRRK2* mutation status, or sex (Fig. 3b). In 60 of 81 CSF samples analyzed, hemoglobin levels were below the limits of reliable detection using our ELISA platform (< 2 pg mL^-1^). Of the remaining samples with measured hemoglobin, there was no correlation between pS1292-LRRK2 / flotillin-1 or other protein measurements (Spearman R <0.1, all *p*>0.4). Two CSF specimens had particularly high hemoglobin levels >200 pg mL^-1^, but these samples had average pS1292-LRRK2, total LRRK2, and flotillin-1 protein levels. These results demonstrate that we could readily measure pS1292-LRRK2 in CSF exosome fractions in a large biobanked series, although levels were not different in LRRK2 mutation carriers despite the robust differences we could observe in urine collected in the same clinic visit in the same subjects.

**Fig. 4:**
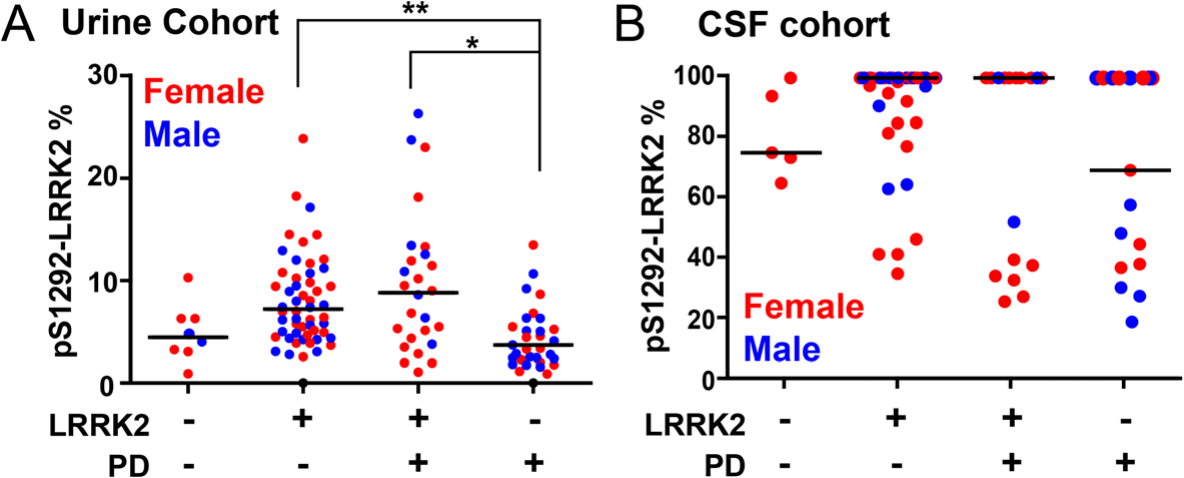
pS1292-LRRK2 levels in urine and saturation in CSF Absolute levels of pS1292-LRRK2 were normalized to total LRRK2 protein to derive a ratio of the percent of total LRRK2 that is autophosphorylated. **a** Scatter plots showing percent pS1292-LRRK2 from urinary exosomes (N=132, blue: male, red: female). **b** Scatter plots showing percent pS1292-LRRK2 from CSF exosomes (N=81, blue: male, red: female). Values above 100% phosphorylation were considered within technical error of detection of full saturation and plotted as 100% for visualization purposes only and not for statistical analysis. Unadjusted absolute values of pS1292-LRRK2 are given in Fig. 5. Bars show median values. ***p-value<0.001, **p-value<0.01, *p-value<0.05, ns: *p*-value>0.05. *p* value between groups were calculated using Dunn’s multiple comparison test (**a** and **b**)

We hypothesized that the reason pS1292-LRRK2/flotillin-1 levels in CSF could not identify LRRK2 mutation carriers was related to the very high proportion of pS1292-LRRK2 in CSF and thus possible ceiling effects inherent to limited substrate (i.e., total LRRK2 protein). We therefore defined the percent of LRRK2 phosphorylated at the 1292 residue in each urine and CSF exosome sample. As expected, we found an elevated proportion of pS1292-LRRK2 in urinary exosomes in LRRK2 mutation carriers compared to non-carriers (7.3% of total LRRK2 protein in mutation carriers versus 4.2% in non-carriers, *p*<0.0001, Fig. 4a). Breaking these groups according to sex, consistent with pS1292-LRRK2/Tsg101 measurements, male carriers with PD had significantly higher phosphorylation at the 1292 residue than carriers without PD (11.5% versus 6.4%, respectively, *p*=0.0239 Fig. 4a2). Samples from females with a LRRK2 mutation and PD again showed a slightly lower percent pS1292-LRRK2 than mutation carriers without PD, although the difference was not significant (6.5% versus 9.4%, respectively, p=0.26, Fig. 4a3).

These analyses in CSF pS1292-LRRK2 levels revealed an unexpected ceiling effect in many samples (Fig. 4). Of the 22 CSF samples from non-LRRK2 mutation carriers, 50% (11 of 22) demonstrated >95% phosphorylation, whereas in LRRK2 mutation carriers, 38 of 57 CSF (67%) samples demonstrated >95% phosphorylation. Stratification of samples that have near-saturated pS1292-LRRK2 (>95%) from those with lower levels (<95%) also did not successfully separate *LRRK2* mutation carriers from non-carriers (chi-square 1.872, p=0.17) or reveal a correlation with clinical scales for PD severity.

### pS1292-LRRK2 correlates with total LRRK2 protein, but urine levels do not predict CSF levels.

Evidence both *in vitro* in primary cultured cells, as well as in rodents and monkeys treated with LRRK2 kinase inhibitors, suggests that LRRK2 kinase activity may enhance protein stability and decrease LRRK2 protein turnover [9, 23, 26, 32]. In considering the absolute amounts of protein on a volumetric basis with no normalization to other exosome proteins, in the average mL of CSF, there is ~120 pg of LRRK2 harbored in exosome fractions, with only a slightly lower amount of pS1292-LRRK2, whereas in urine there is much less pS1292-LRRK2 protein but similar total LRRK2. In both urine and CSF, a strong positive correlation exists between pS1292-LRRK2 and total LRRK2 (Spearman’s rho 0.76 and 0.38, respectively, p<0.0001 for both), giving further confidence that the pS1292-LRRK2 signal, always measured on different immunoblots than total LRRK2, is authentic in both urine and CSF.

**Fig. 5:**
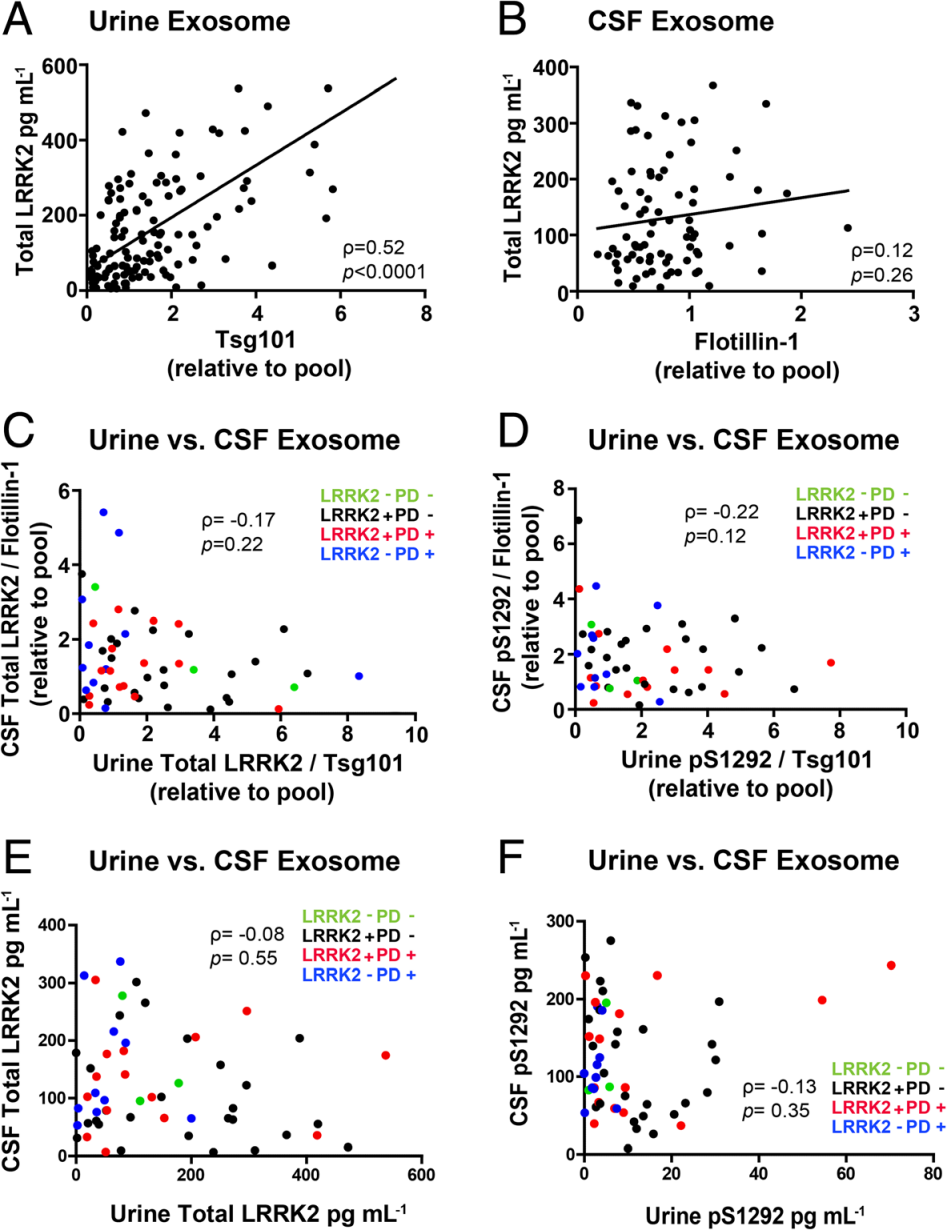
Comparing urine and CSF exosomal LRRK2-expression and phosphorylation profiles. **a** Correlation of total LRRK2 and TSG101 in urine (N=132), and (**b**) total LRRK2 and flotillin-1 in CSF (N=81). **c** Correlation of total exosomal LRRK2 protein in CSF and urine, and (**d**) pS1292-LRRK2 in CSF and urine. Without adjusting for housekeeping exosome protein concentrations, correlation for (**e**) total LRRK2 protein and (**f**) pS1292-LRRK2 protein in CSF and urine. Samples were matched to the same clinic visit. (N=56, green: LRRK2- PD-, black: LRRK2+ PD-, red: LRRK2+ PD+, blue: LRRK2- PD+). Spearman's rank correlation coefficients are shown with corresponding *p* values

While more LRRK2 protein positively predicts more pS1292-LRRK2 protein in both CSF and urine exosomes, we wondered whether more exosomes in general in the biofluids predicts more LRRK2 protein that we can measure. In urine, there was a positive correlation between LRRK2 protein concentration and Tsg101 levels (Spearman’s rho 0.52, p<0.0001), showing the Tsg101 may be in the same exosome population as LRRK2, consistent with previous observations [7]. In addition, there is an overall increase in LRRK2 protein in males compared to females (2.61 in males versus 1.64 in females, p = 0.003), consistent with recent observations in a cohort from Birmingham, Alabama [8]. In contrast, in CSF exosomes, where Tsg101 protein was not reliably detected, we found that flotillin-1 poorly correlated with LRRK2 protein levels (Fig. 5). These results suggest that the majority of the pool of flotillin-1 positive exosomes may not be LRRK2 positive. Further, levels of LRRK2 normalized to Tsg101 in urine and levels of LRRK2 normalized to flotiiln-1 in CSF did not correlate with one another in subjects from our cohort (Fig. 5c, d). Irrespective of other exosomal proteins, absolute levels of both total LRRK2 protein as well as pS1292-LRRK2 protein in CSF and urine exosome fractions also did not correlate (Fig. 5e, f). These results suggest that the parental cells shedding LRRK2-positive exosomes within an individual have differential regulation of LRRK2 expression and regulation of autophosphorylation with respect to LRRK2 mutation status and PD diagnosis.

## Discussion

Our results include three key observations. First, we provide additional support for pS1292-LRRK2 in exosomes as a surrogate for LRRK2 kinase activity, with the caveat that not all LRRK2-expressing cells like macrophages may contribute LRRK2 protein equally to the exosome pool. Second, we can demonstrate in a novel Norwegian cross-sectional cohort that pS1292-LRRK2 levels are elevated in urinary exosomes from G2019S-*LRRK2* mutation carriers compared to non-carriers, in both males and females. These results support data from model systems that suggest kinase activating effects of the prevalent G2019S-*LRRK2* mutation. According to our cohort, different than in males, in females there is no predictive value of exosome pS1292-LRRK2 levels in PD diagnosis, potentially owing to the overall lower LRRK2 levels in females versus males and overall positive correlation of pS1292-LRRK2 to total LRRK2 protein in all subjects [8]. Third, we provide evidence that LRRK2 autophosphorylation at S1292 may be much higher in brain-derived exosomes compared to exosomes purified from urine and other cellular sources. Although an important limitation of the study is that lack of male healthy controls that donated CSF, the high and often saturated pS1292-LRRK2 appears to be common to both male and female subjects that did donate CSF. Since cellular and exosome levels of pS1292-LRRK2 correlate, these results may show that LRRK2 activity is much higher in brain cells that shed exosomes into CSF than in the peripheral cells that contribute to urinary exosome pools. Overall these results provide some of the first insights into the putative pathological pS1292-LRRK2 species in the brain and periphery in a clinical population.

Despite the link between LRRK2 and PD that has been known for over twelve years, and the implication of LRRK2 kinase activity in PD susceptibility, few studies have yet measured LRRK2-kinase dependent phosphorylation in clinical samples. This is because LRRK2 kinase substrates have been very difficult to identify and tend to be poorly expressed. In our transfected cells, and in primary macrophages that express very high LRRK2 protein, the amount of autophosphorylated LRRK2 is much lower than in our clinical samples. We speculate that part of the difficulty in finding LRRK2 substrates can be explained by low or no kinase-active associated with LRRK2 protein when expressed in some model systems. So far, two LRRK2 kinase substrates have been identified that increase in abundance with pathogenic LRRK2 mutations, decrease in abundance with LRRK2 kinase inhibition, and serve as substrates *in vitro*: LRRK2 protein itself via pS1292 [23] in *cis*-autophosphorylation, and other small-GTPase Rabs that share similarity with the LRRK2 GTPase domain that include Rab10 and Rab8 [26]. Unfortunately, we were unable to detect robust levels of Rab10 or Rab8 in exosome fractions from either urine or CSF. However, our results show that pS1292-LRRK2 can be detected and measured in both urine and CSF exosomes.

Exosome-derived LRRK2 and pS1292-LRRK2 have been analyzed using traditional immunoblotting techniques for the current study. It must be noted that measurements of exosome-derived LRRK2 has not been replicated in orthogonal assays like ELISA, single-particle detection, or the LC-MS assay used here to measure recombinant pS1292-LRRK2. Caution should be used in data interpretation until multiple types of scalable assays demonstrate consistent congruency in measures related to LRRK2 in biofluids. In the meantime, well-characterized recombinant protein standards that help define absolute concentrations have demonstrated utility to interpretation.

There are several lines of evidence that the pS1292-LRRK2 signal we measured here is authentic: First, the pS1292-LRRK2 signal disappears with LRRK2 kinase inhibitors or mutation of Ser1292 to alanine, and is increased in abundance with pathogenic LRRK2 mutations in both model systems and urinary exosomes. Second, the pS1292-LRRK2 signal, as in CSF exosomes, is dependent on phosphorylation, since phosphatase treatment of membranes ablates signal. Third, the pS1292-LRRK2 signal is strongly correlated with total LRRK2 signal, and the LRRK2 antibodies used here are raised against epitopes distant from the pS1292-LRRK2 epitope. While only one antibody epitope can be used for pS1292-LRRK2 detection, several high-quality total LRRK2 monoclonal antibodies are available and the signal these generate are perfectly correlated in the subject exosome lysates, indicating they detect the same protein (Additional file 1: Figure S3).

The rationale for LRRK2 protein detection in exosome fractions began with our initial microscopy studies where we and others could observe a small proportion of LRRK2 protein associated with intra-luminal vesicles in multi-vesicular bodies [3, 7]. First in transfected HEK293 cells, we could demonstrate that LRRK2 protein can be measured in extracellular fractions enriched in exosomes, and the pS1292-LRRK2 levels are correlated with the cellular levels. As compared to measurements of other proteins in the extracellular milieu, exosomes provide an ideal source for protein since the robust protective lipid-bilayer shields the exosome proteins from extracellular enzymes that might include phosphatases and proteases [28]. Further, the exosome proteome with hundreds of proteins is extremely reduced in complexity compared to the complexity of tissue proteomes with thousands of proteins [22, 27].

While exosomes can be isolated from all known biological fluids, we and others have not *a priori* been able to predict which proteins and modifications can be measured in exosomes based on the cellular constituency of the parental sources. For example, we have been so-far unable to reliably detect LRRK2 expression in exosomes isolated from serum (Additional file 1: Figure S5), despite the abundance of LRRK2 expression in circulating leukocytes. This result, in combination with measures of hemoglobin in CSF, suggest that LRRK2 protein in CSF we measured is unlikely to be related to blood contamination. There is yet little information on the origins of the diversity of microvesicle subpopulations in clinical samples like CSF and urine. Thus, the types of cells in the body that contribute LRRK2-positive exosomes in the periphery in urine and in the brain in CSF are unknown. Furthermore, we are unable to assess the degree of variability, regarding to total exosome number, size, and distribution between individuals and groups because we were unable to perform nanoparticle tracking in every sample due to limitations in sample volumes available. Future studies will be critical in establishing the normal biology of different subsets of exosomes and microvesicles. In this study and our past studies, pS1292-LRRK2 levels in urinary exosomes have been robust in separating LRRK2 mutation carriers from non-carriers [6, 8]. Given the striking differences in the amount of LRRK2 autophosphorylation that occurs in the brain versus peripheral exosomes, LRRK2 kinase activity does not appear hard-coded intrinsically within the protein as has been suspected, but regulated by an external network of factors. This observation appears to hold true in mice as well, since LRRK2 protein isolated from the lung has much less kinase activity than the same amount of LRRK2 isolated from the brain [16]. This may reflect a unique aspect of LRRK2 biology in the human brain that may be important for PD susceptibility.

Several direct applications and extensions based on our work here can be envisaged. First, in an independent cohort with subjects on a different ethnic background (Norwegian), in males, but not females, we could confirm the potential of pS1292-LRRK2 levels in urine as a candidate biomarker for PD susceptibility, where non-manifesting subjects tended to have lower pS1292-LRRK2 levels. Notably, our sample size of male G2019S carriers with PD was half that of our first study (N=8 here versus N=16 in our past study) and a difference was still detected. Thus, urinary exosome pS1292-LRRK2 levels may help identify male mutation carriers at the highest risk for PD onset, together with other emerging biomarkers. Future studies using highly specific, sensitive and scalable orthogonal methods of LRRK2 and phosphorylated LRRK2 quantification are critically needed for the translation of our results to clinical utility.

## Conclusions

The G2019S-LRRK2 mutation upregulates LRRK2-kinase activity-dependent autophosphorylation at Ser1292 in exosomes captured from peripheral and brain-derived exosomes. LRRK2 protein in brain exosomes may be much more active than in the periphery in most subjects.

## Additional files

Additional file 1: Supplemental figures. (PDF 5036 kb)

## Abbreviations

COR: C-terminal of ROC
CSF: Cerebral spinal fluid
H&Y: Hoehn & Yahr scale
LEDD: L-dopa equivalent daily dose (LEDD)
LRRK2: Leucine rich repeat kinase 2
MoCA: Montreal Cognitive Assessment
mS&E: Schwab & England scale
PD: Parkinson’s disease
ROC: Ras-of-complex
UPDRS: Unified Parkinson’s Disease Rating Scale
