# Supplementary material for: Elevated LRRK2 autophosphorylation in brain-derived and peripheral exosomes in LRRK2 mutation carriers
Source: Acta Neuropathol Commun. 2017 Nov 22;5:86. doi: 10.1186/s40478-017-0492-y (PMC5700679; doi:10.1186/s40478-017-0492-y)
Supplement: Supplementary file 1 — Supplemental figures. (PDF 5036 kb) [file 40478_2017_492_MOESM1_ESM.pdf]

Elevated LRRK2 autophosphorylation in brain-derived and peripheral exosomes in LRRK2 mutation carriers

Shijie Wang[1], Zhiyong Liu[1], Tao Ye[2], Omar S. Mabrouk[2], Tyler Maltbie[1], Jan Aasly[3]*, Andrew B. West[1]*

[1] Center for Neurodegeneration and Experimental Therapeutics, Department of Neurology, University of Alabama at Birmingham, Birmingham, AL 35233 USA

[2] Biogen, Discovery and Early Development Biomarkers, Cambridge, MA 02142, USA

[3] Dept of Neurology, St. Olavs Hospital, and Dept of Neuroscience, Norwegian University of Science and Technology, 7030 Trondheim, Norway

Supplemental Figures

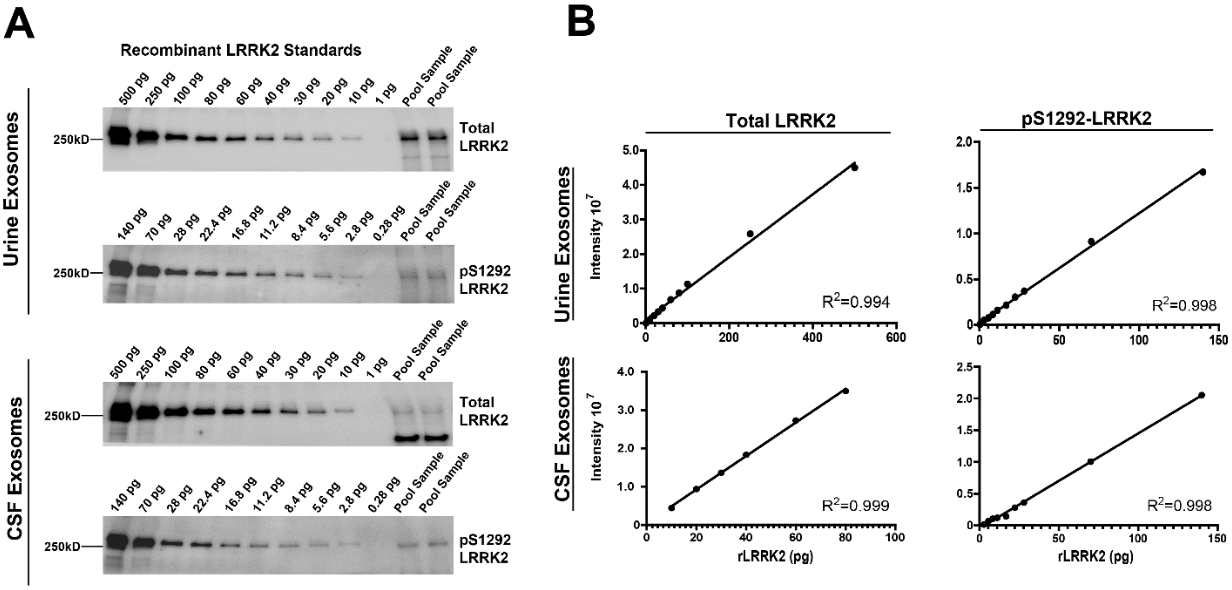

Supplement figure 1. Standard curves in urine and CSF pooled samples.
(A) Immunoblots of pooled exosome lysates from urine and CSF cohort, along with indicated concentration of recombinant LRRK2 standards in the possible range of intensities associated with clinical samples. Blots were probed with antibodies against total LRRK2 and pS1292-LRRK2. Intensities were quantified and plot as standard curve **(B)**. Pearson's R is shown.

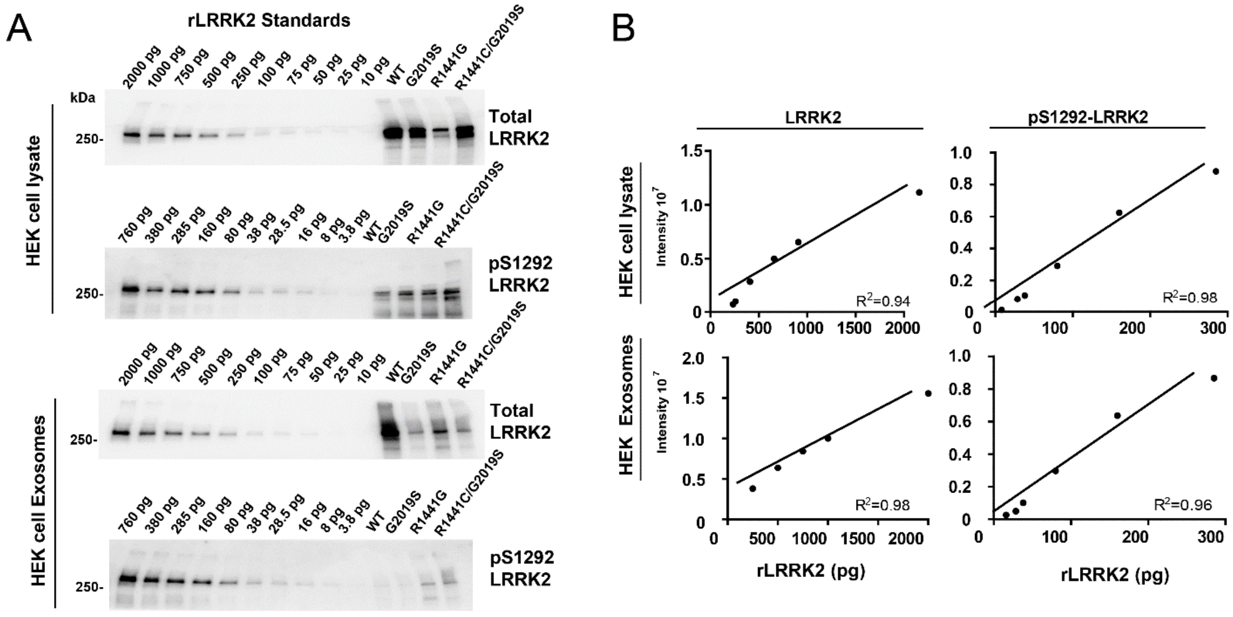

Supplement figure 2. Standard curves in urine and CSF pooled samples.
(A) Immunoblots of HEK cell lysates and HEK cell exosome lysates, along with indicated concentration of recombinant LRRK2 standards in the possible range of intensities. Blots were probed with antibodies against total LRRK2 and pS1292-LRRK2. Intensities were quantified. **(B)** Pearson's R is shown.

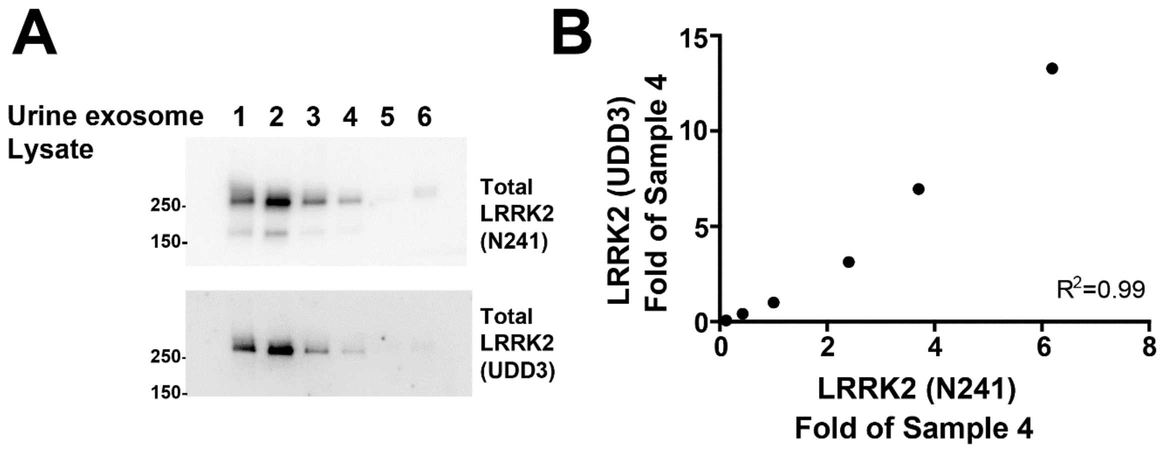

Supplement figure 3. Cross validation of total LRRK2 antibodies using urine exosome samples. (A) Immunoblots of six selected urinary exosome lysates, selected at random, from the sample cohort. Blots probed with two total LRRK2 monoclonal antibodies targeting either the N-terminus (UDD3) or the C-terminal half (N241) of LRRK2 showed similar levels of LRRK2. Intensities were quantified and plotted normalized to the intermediate LRRK2-expressing sample 4. **(B)**. Pearson's R is shown.

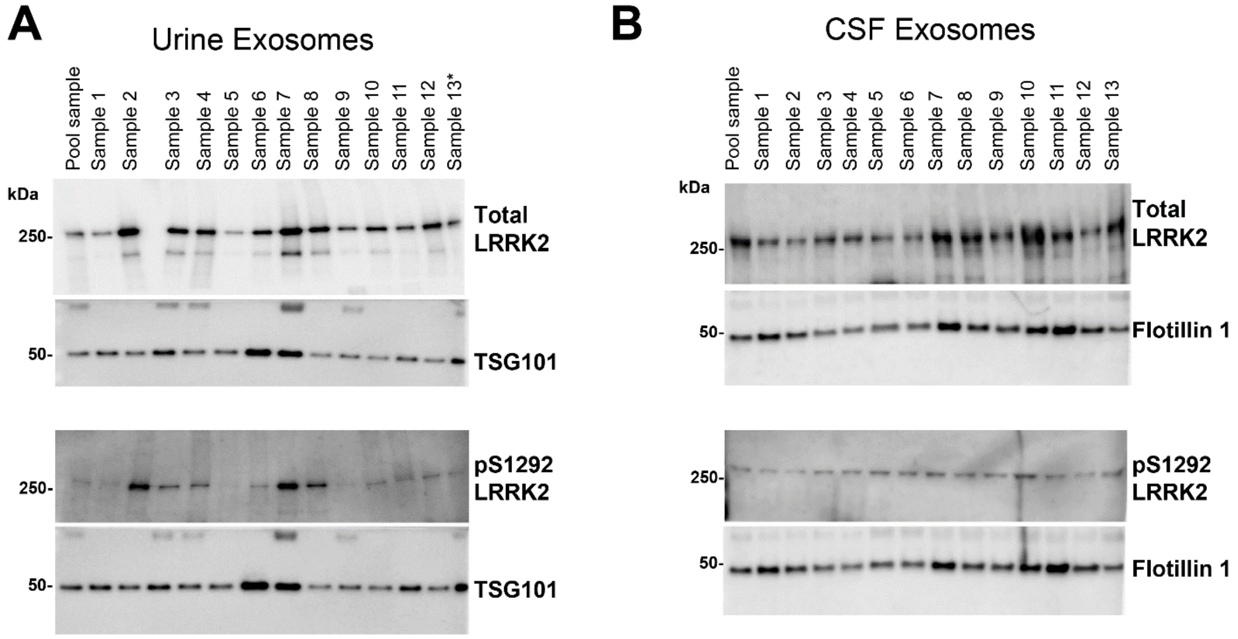

Supplement figure 4. Representative immunoblots of pS1292-LRRK2, total LRRK2, and exosome house-keeping proteins TSG101 and flotillin 1 in urine and CSF exosome samples. (A) Representative images of an immunoblot run are displayed, and these signals analyzed via digital recording of luminescence prior to sample unblinding. 13 exosome lysate from the urine sample cohort or **(B)** CSF cohort are shown. Samples were loaded into two different blots with the bottom halves probed with housekeeping proteins, and the top half probed with LRRK2 monoclonal antibodies. An * denotes a sample that failed quality control (see Methods) and was not included in the analysis (Sample 13 from urinary exosome runs).

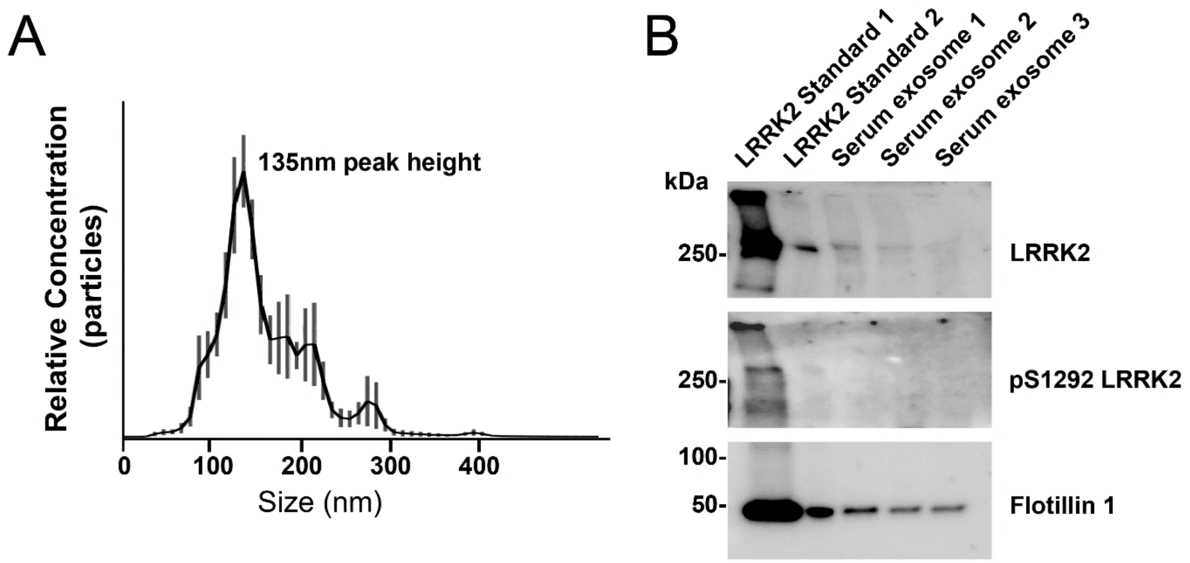

Supplement figure 5. Isolation and characterization of exosomes from serum samples.
(A) Representative nanoparticle tracking analysis of the exosome-enriched fraction from serum.
(B). Immunoblots of three serum exosome-enriched pellet lysates together with recombinant LRRK2 protein control added into HEK293 lysates (standard 1 and standard 2).
